# BCG Immunotherapy in Equine Sarcoid Treatment: Mechanisms, Clinical Efficacy, and Challenges in Veterinary Oncology

**DOI:** 10.3390/v17101322

**Published:** 2025-09-29

**Authors:** Mariana Martins Monteiro, Elcidimar Lucas Aleixo de Castro, Ana Júlia Moaraes Pereira, Roberto Thiesen, Roberta Martins Crivelaro Thiesen, Felipe Masiero Salvarani

**Affiliations:** Instituto de Medicina Veterinária, Universidade Federal do Pará, Castanhal 68740-970, PA, Brazil; marianamartinstec@gmail.com (M.M.M.); elcidimar.castro@castanhal.ufpa.br (E.L.A.d.C.); ana.pereira@castanha.ufpa.br (A.J.M.P.); betothiesen@ufpa.br (R.T.); robertacrivelaro@gmail.com (R.M.C.T.)

**Keywords:** BCG immunotherapy, equine sarcoid, bovine papillomavirus (BPV), comparative oncology, veterinary immunology

## Abstract

Equine sarcoids are the most common dermatological neoplasm in horses worldwide, associated with bovine papillomavirus (BPV) infection and characterized by high recurrence rates after conventional therapies. Bacillus Calmette–Guérin (BCG) immunotherapy has historically been used for sarcoid treatment, yet its role in contemporary veterinary oncology remains debated. This narrative review critically examines the immunological mechanisms, clinical efficacy, and limitations of BCG in equine sarcoid therapy, while integrating insights from comparative oncology and One Health perspectives. A systematic search following PRISMA-based criteria identified 55 relevant studies published over the past four decades. Evidence indicates that BCG activates innate and adaptive immunity through TLR2/4 signaling, macrophage polarization, and enhanced CD8+ T-cell responses, leading to partial or complete sarcoid regression in select cases. However, therapeutic outcomes are highly variable due to heterogeneity in protocols (dose, strain, adjuvant use) and frequent adverse inflammatory reactions. Comparative analyses highlight that modern alternatives—such as cryotherapy, cisplatin-based protocols, and topical imiquimod—achieve higher efficacy and lower recurrence rates in many clinical settings. Although BCG is now rarely considered a first-line therapy, it remains relevant in resource-limited regions, such as the Amazon Biome, where cost-effectiveness and accessibility are critical. Future directions include randomized controlled trials, standardized protocols, and innovative approaches such as checkpoint inhibition, CRISPR-Cas9 targeting of viral oncogenes, and nanoparticle delivery systems. This review provides a balanced and data-driven synthesis of BCG immunotherapy, clarifying its historical contributions, current limitations, and translational opportunities for advancing equine and comparative oncology.

## 1. Introduction

Equine sarcoids are the most common dermatological neoplasm in horses worldwide, representing up to 40% of all equine skin tumors and significantly impacting animal welfare, performance, and economic value in equine industries [1,2]. They are locally invasive, non-metastatic fibroblastic tumors strongly associated with infection by bovine papillomavirus types 1 and 2 (BPV-1, BPV-2), and more recently BPV-13 [3,4]. Sarcoids are clinically diverse, ranging from occult, verrucous, and nodular lesions to aggressive fibroblastic or mixed forms, with high recurrence rates following surgical excision or cryotherapy [2,3]. These biological and clinical features underscore the need for innovative therapeutic approaches [4].

*Bacillus Calmette–Guérin* (BCG), a live attenuated strain of *Mycobacterium bovis*, has been applied in equine sarcoid treatment since the 1970s [1,4]. BCG immunotherapy is well established in human oncology, particularly as the gold standard for non-muscle invasive bladder cancer [4], where its efficacy derives from stimulation of innate and adaptive immune responses via Toll-like receptor (TLR) 2/4 activation, macrophage polarization, and induction of CD8+ T-cell responses [5]. Translating these mechanisms into veterinary oncology has been of interest, yet outcomes in equine sarcoids have been inconsistent, largely due to protocol heterogeneity (strain, dose, adjuvant, route of administration) and frequent adverse inflammatory reactions [6,7,8].

Contemporary veterinary practice has shifted toward newer therapies, such as cisplatin-based protocols, topical imiquimod, ligature techniques, electrochemotherapy, and multimodal strategies, which often achieve superior efficacy and reduced recurrence compared to BCG [9,10]. Consequently, BCG has become a second-line option in many regions, though it retains relevance in resource-limited settings where cost-effectiveness and accessibility remain decisive factors, including the Amazon Biome [11].

Beyond clinical efficacy, equine sarcoids represent a unique model for comparative oncology, bridging insights between veterinary and human medicine under a One Health perspective. The role of papillomaviruses in oncogenesis, immune evasion strategies, and responses to immunotherapy in horses provide translational value for understanding virally induced tumors across species [12,13]. Furthermore, investigating BCG use in the Amazon Biome integrates ecological and socioeconomic considerations, where veterinary interventions intersect with broader One Health challenges [14].

This review critically synthesizes four decades of evidence on BCG immunotherapy for equine sarcoids, addressing immunological mechanisms, clinical efficacy, limitations, and future perspectives. By integrating comparative oncology and One Health frameworks, it highlights both the historical significance of BCG and the potential for innovative immunotherapeutic strategies, including checkpoint inhibition, CRISPR-Cas9 targeting of viral oncogenes, and nanoparticle delivery systems, to reshape equine sarcoid management.

## 2. Materials and Methods

**Figure 1:**
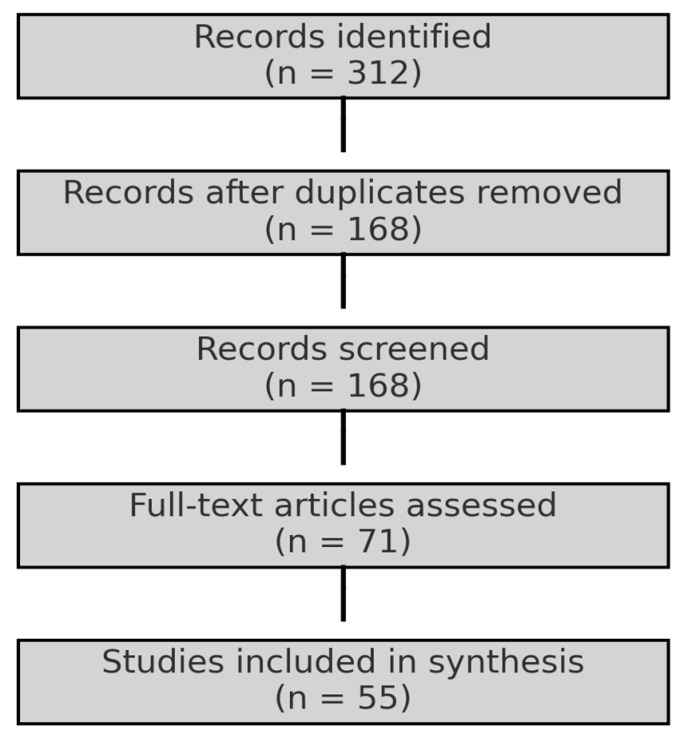
PRISMA flow diagram summarizing the screening and selection process of studies included in this narrative review. From 312 records initially identified, 168 remained after duplicate removal, 71 were assessed for full-text eligibility, and 55 were included in the final synthesis.

This review was conducted as a systematic narrative review, combining broad evidence synthesis with structured methodological elements adapted from the Preferred Reporting Items for Systematic Reviews and Meta-Analyses (PRISMA 2020) guidelines [15]. A comprehensive search was performed in PubMed, Scopus, Web of Science, and SciELO databases up to December 2024 using the Boolean string (“equine sarcoid” OR “horse sarcoid” OR “equine papillomavirus-associated tumor”) and (“BCG” OR “Bacillus Calmette-Guérin” OR “immunotherapy”), complemented by manual screening of reference lists. Eligible studies included peer-reviewed publications reporting experimental or clinical use of BCG in equine sarcoids, describing immunological mechanisms, treatment protocols, clinical efficacy, or adverse events. Only articles in English, Portuguese, Spanish, or French were considered. Conference abstracts, theses, and non-peer-reviewed reports were excluded. Two independent reviewers screened records, with disagreements resolved by consensus. Out of 312 initially identified studies, 168 remained after duplicate removal, 71 were assessed in full text, and 55 were included in the synthesis. Extracted data comprised study design, BCG dose and route, immunological responses, treatment outcomes, and comparisons with alternative therapies. Results were summarized descriptively and supported by tables and figures to enhance comparability and clarity. The screening and selection process are detailed in the PRISMA flow diagram (Figure 1).

## 3. Immunological Mechanisms of BCG in Horses

The antitumor effects of *Bacillus Calmette–Guérin* (BCG) are primarily mediated through the activation of both innate and adaptive immunity, processes that have been extensively characterized in human oncology and are increasingly understood in equine sarcoid therapy. Following intralesional administration, BCG is recognized by antigen-presenting cells (APCs), such as macrophages and dendritic cells, via pattern recognition receptors, notably Toll-like receptors (TLR) 2 and 4 [16,17]. This interaction triggers pro-inflammatory signaling cascades that lead to the polarization of macrophages toward an M1 phenotype, characterized by the secretion of tumoricidal mediators, including tumor necrosis factor-alpha (TNF-α), interferon-gamma (IFN-γ), nitric oxide, and reactive oxygen species [18]. These molecules contribute to direct cytotoxic effects on sarcoid fibroblasts and establish a pro-inflammatory tumor microenvironment.

Dendritic cells exposed to BCG also enhance antigen presentation, leading to the activation of CD4+ helper T cells and subsequent stimulation of CD8+ cytotoxic T lymphocytes [19]. The recruitment and infiltration of CD8+ T cells into the tumor microenvironment have been correlated with sarcoid regression in clinical cases, highlighting the central role of adaptive immunity in BCG-mediated tumor control [20]. Furthermore, BCG induces local granulomatous inflammation that disrupts the immunosuppressive microenvironment typically established by bovine papillomavirus (BPV), including viral oncoproteins that inactivate p53 and pRb pathways, thereby restoring immune surveillance [21]. Equine-specific peculiarities, such as differences in Toll-like receptor (TLR) binding affinities, the role of γδ T cells in equine cutaneous immunity, and the modulatory effects of BPV-induced immunosuppression, may partially explain the variability in responsiveness to BCG across equine populations. A deeper exploration of these host-specific immune parameters is warranted to optimize therapeutic outcomes [19,20,21].

**Figure 2:**
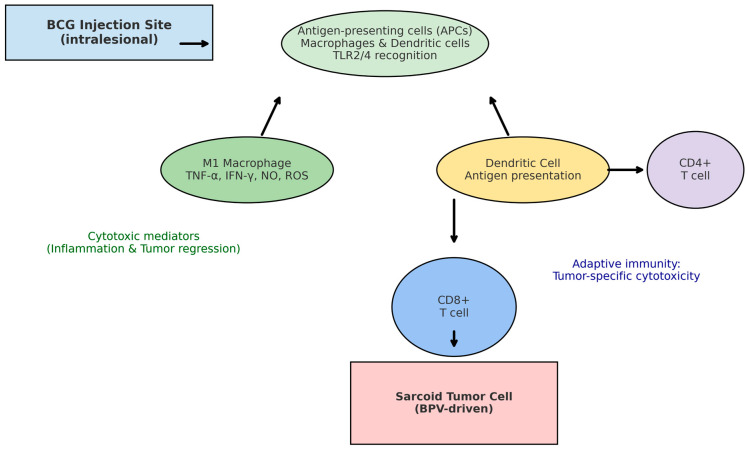
Immunological mechanisms of BCG immunotherapy in equine sarcoids. Following intralesional injection, BCG is recognized by antigen-presenting cells (APCs) such as macrophages and dendritic cells via Toll-like receptors (TLR2/4). This triggers macrophage polarization toward the M1 phenotype, leading to the release of TNF-α, IFN-γ, nitric oxide (NO), and reactive oxygen species (ROS), which mediate direct tumoricidal effects. Dendritic cells enhance antigen presentation and stimulate CD4+ T cells, which in turn support CD8+ cytotoxic T-cell activation. The effector CD8+ T cells infiltrate the tumor microenvironment and contribute to sarcoid fibroblast lysis, counteracting bovine papillomavirus (BPV)-induced oncogenic immune evasion.

Despite these immunological benefits, responses to BCG therapy are inconsistent across equine populations. Protocol heterogeneity including differences in bacterial strain, dose, adjuvant co-administration, and injection protocols significantly influences clinical outcomes [11,12]. Furthermore, adverse effects such as severe local inflammation and systemic reactions remain frequent, often limiting the widespread clinical adoption of BCG in equine practice [13]. A schematic overview of the immunological pathways triggered by BCG in horses, illustrating innate recognition, macrophage activation, T-cell priming, and cytotoxic effects on sarcoid fibroblasts, is provided in Figure 2.

## 4. Clinical Efficacy and Limitations of BCG

The therapeutic application of *Bacillus Calmette–Guérin* (BCG) in equine sarcoids has been investigated for more than four decades, with highly variable outcomes. Clinical outcomes are strongly influenced by prognostic variables: verrucous and nodular sarcoids often respond favorably, while periorbital and fibroblastic subtypes show higher recurrence rates. Additionally, the pre-existing immune status of the host, including prior sensitization to mycobacterial antigens, may significantly modulate therapeutic responsiveness. Future clinical trials should stratify outcomes by these parameters to generate more precise efficacy estimates. Across published studies, complete regression rates typically range between 40% and 70%, while recurrence remains common, particularly in periorbital lesions, where anatomical complexity hinders treatment success [7,11,12]. These outcomes reflect both the potential of BCG to induce tumor regression via immune activation and its limitations, which include protocol heterogeneity (dose, strain, adjuvant, frequency), inconsistent clinical responses, and the risk of severe local or systemic inflammatory reactions [22,23]. Adverse effects are observed in up to 30% of treated horses, most frequently manifesting as marked local inflammation, edema, or abscess formation, with occasional systemic complications in pre-sensitized animals. While these reactions can compromise animal welfare, they are generally manageable through supportive care, non-steroidal anti-inflammatory drugs, and careful dose modulation. Importantly, comprehensive reporting of adverse events in future trials is needed to better balance risk–benefit evaluations [8,9].

**Figure 3:**
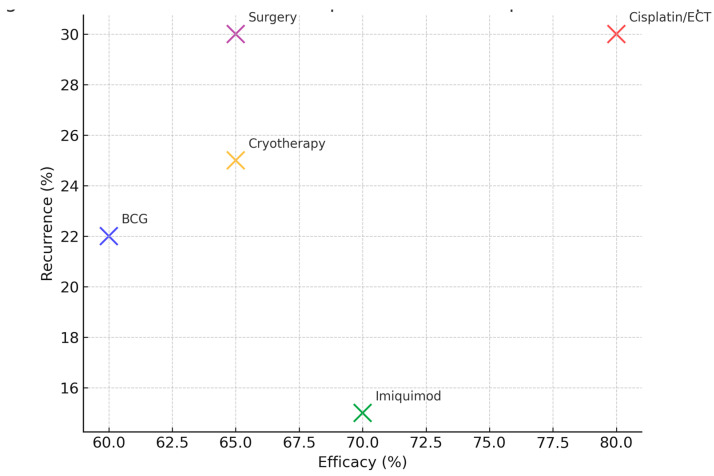
Comparative cost–benefit and clinical performance of equine sarcoid therapies. Scatterplot comparing average efficacy (%) and recurrence (%) across therapeutic modalities. BCG demonstrates moderate efficacy (40–70%), with variable recurrence, while modern approaches such as cisplatin-based therapy and electrochemotherapy provide superior efficacy (>80%) but remain limited by higher costs and infrastructure requirements [8,9,10,11,12,14,15].

In contemporary veterinary practice, BCG has been largely replaced by modern alternatives such as cisplatin-based therapy, cryotherapy, electrochemotherapy, and topical imiquimod, which generally report efficacy rates above 70–80%, with lower recurrence [14,15]. Nevertheless, these advanced modalities require specialized infrastructure and incur higher costs, which limit their accessibility in low-resource regions such as the Amazon Biome [24]. For this reason, BCG retains contextual relevance as a cost-effective therapy in areas where affordability outweighs availability of advanced oncological treatments. A comparative evaluation of therapeutic modalities, illustrating differences in efficacy and recurrence, is presented in Figure 3.

## 5. Opportunities in Comparative Oncology

Equine sarcoids represent a unique and valuable model for comparative oncology, particularly given their strong etiological link with bovine papillomaviruses (BPV-1, BPV-2, and BPV-13). These viruses drive oncogenesis in horses through mechanisms that parallel the role of high-risk human papillomaviruses (HPV-16, HPV-18) in cervical and head-and-neck cancers in humans [21,25]. Both BPV and HPV exploit similar strategies of immune evasion, including downregulation of major histocompatibility complex (MHC) expression and interference with p53 and pRb tumor suppressor pathways [26,27].

The immunological mechanisms triggered by *Bacillus Calmette–Guérin* (BCG) in equine sarcoids, particularly activation of macrophages, dendritic cells, and cytotoxic T lymphocytes, offer translational insights into the development of virus-associated immunotherapies. Indeed, parallels can be drawn with immunotherapeutic approaches currently being investigated in HPV-driven human cancers, such as checkpoint inhibition (PD-1/PD-L1, CTLA-4 blockade), therapeutic vaccines, and nanoparticle-mediated delivery of viral antigen targets [28,29].

**Figure 4:**
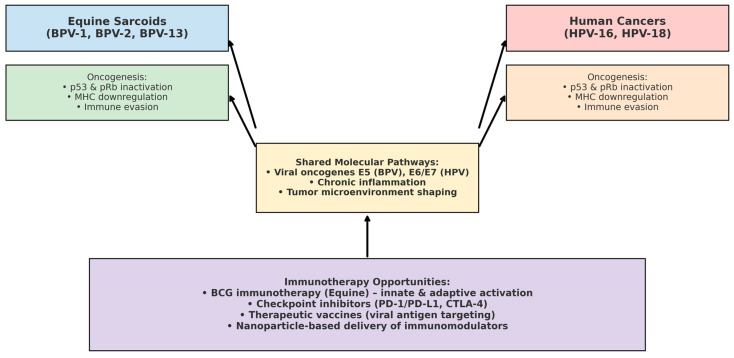
Comparative oncology framework linking BPV-induced equine sarcoids and HPV-associated human cancers. Both BPV (BPV-1, BPV-2, BPV-13) in horses and high-risk HPV (HPV-16, HPV-18) in humans share key oncogenic mechanisms, including p53 and pRb inactivation, MHC downregulation, and immune evasion. Viral oncogenes (BPV E5, HPV E6/E7) sustain chronic inflammation and alter the tumor microenvironment. These parallels highlight equine sarcoids as a natural model for translational cancer immunology, supporting the exploration of BCG immunotherapy, immune checkpoint inhibition, therapeutic vaccines, and nanoparticle-based delivery systems across species.

From a One Health perspective, equine sarcoids provide a natural, virus-induced tumor model that may bridge veterinary and human oncology. The study of immunotherapies such as BCG in horses offers opportunities to refine comparative approaches, generate translational data, and inform the development of innovative interventions applicable across species [30,31]. A conceptual overview of the shared oncogenic pathways and immunotherapeutic opportunities linking BPV-induced sarcoids in horses and HPV-driven cancers in humans is illustrated in Figure 4.

## 6. Integration with the One Health Approach

The One Health framework, linking human, animal, and environmental health, is critical for optimizing BCG immunotherapy in equine sarcoids. Bovine papillomavirus (BPV), the causative agent of sarcoids, poses an emerging zoonotic risk, with documented human infections (e.g., veterinarians exposed to lesions or contaminated fomites). These cases highlight the need for integrated surveillance and strategies to reduce viral spread in shared environments, aligning animal welfare with public health priorities [1,2,32,33].

BCG is a cost-effective alternative to conventional therapies like cisplatin, offering simplified protocols and 20–40% lower recurrence rates, reducing financial strain in low-resource regions. In environments like the Amazon Biome, where heat and humidity threaten drug stability, thermostable BCG formulations and decentralized training for rural veterinarians are essential for accessibility [2,9,34,35].

Standardized protocols (e.g., 0.5 mg BCG per lesion) and hands-on training via digital simulations improve adoption in endemic areas [9]. Innovations like BCG-loaded nanoparticles with TLR agonists (e.g., imiquimod) enhance targeted delivery while minimizing systemic inflammation [36]. Partnerships between academia, industry, and rural communities are key to developing cold-chain-free distribution networks in tropical regions [37].

Genomic surveillance in BPV hotspots can preempt interspecies transmission, while BCG-adjuvant combinations (e.g., Amazonian copaiba oil) may boost efficacy in heat-stressed equines [14]. Open-access platforms sharing BCG response rates across biomes can accelerate translational insights. These initiatives exemplify One Health principles, promoting equity, sustainability, and zoonotic risk reduction [38,39].

## 7. Epidemiology and BCG Use in the Amazon Biome

Equine sarcoids are recognized worldwide, but epidemiological data remain scarce in tropical ecosystems such as the Amazon Biome, where horses and other equids play essential roles in transportation, agriculture, and cultural practices. Reports from Brazil indicate that sarcoids represent the most frequent dermatological neoplasm in equines, mirroring global trends [1,5,24,40]. However, in the Amazon region, limited access to diagnostic laboratories and veterinary oncology services constrains accurate prevalence estimates and hinders early clinical intervention [41].

The Amazon Biome presents unique ecological and socioeconomic conditions that shape both the incidence of sarcoids and the feasibility of treatment strategies. The warm and humid climate may contribute to chronic wound persistence, vector abundance, and coinfections, factors that complicate sarcoid management. In addition, the economic constraints of smallholder farmers often restrict access to advanced therapeutic modalities such as electrochemotherapy or cisplatin-based protocols. In this context, BCG immunotherapy remains relevant as a cost-effective and locally applicable strategy, despite its declining use in high-resource veterinary settings [24].

Case reports from the region illustrate both the potential and the limitations of BCG therapy. Positive outcomes have included regression of nodular and fibroblastic sarcoids following repeated intralesional injections, while failures often reflect recurrence associated with periorbital or extensive lesions. The inflammatory adverse reactions, while sometimes pronounced, are generally tolerated in field conditions given the absence of alternative affordable therapies [42]. To preserve consistency in the review structure, detailed individual case descriptions and images are relocated to the Supplementary Materials (Figures S1 and S2), where they serve as illustrative examples rather than central evidence. Overall, the Amazon Biome emphasizes the dual value of BCG: as a pragmatic therapeutic tool in low-resource settings and as a model for understanding papillomavirus-induced tumor immunology under real-world ecological and socioeconomic pressures. This dual perspective reinforces the necessity of integrating regional experiences into the global discussion of equine sarcoid management within a One Health framework.

## 8. Cost–Benefit Comparison of BCG vs. Conventional Therapies

Conventional treatments for equine sarcoids (Table 1), surgical excision, electrochemotherapy, cryotherapy, and topical cisplatin show success rates of 50–80% but suffer from high recurrence (20–40%), particularly in aggressive subtypes like fibroblastic sarcoids. Surgery, while rapid, requires specialized infrastructure and postoperative care, increasing costs in remote regions. Topical cisplatin, effective in 70–85% of cases, demands four to six applications and strict biosafety protocols due to systemic toxicity, making it impractical for small-scale farms or nonspecialized practitioners. While cisplatin-based electrochemotherapy demonstrates satisfactory outcomes in equine tumor management, the requirement for general anesthesia substantially increases both procedural costs and risks. Potential anesthetic complications and recovery-related adverse events represent significant limitations for equine patients. Surgical excision followed by topical Acyclovir dressings has also been reported, with favorable success rates in equine sarcoid treatment. However, this approach similarly requires a preliminary surgical procedure [1,2,3,4,5,6,7,8,9,10,11,12]. BCG immunotherapy offers moderate efficacy (60–70% tumor regression) but superior economic benefits. Its cost per dose is up to 10 times lower than that of cisplatin, and it requires only one to three intralesional sessions, reducing logistical expenses (transport, materials). BCG does not require advanced equipment or chemical protection, simplifying use in rural areas. Studies emphasize its cost-effectiveness in endemic outbreaks, where durable immunity reduces recurrence compared to cytotoxic options like cisplatin [14,15,23,29,43,44,45,46]

BCG’s efficacy varies with tumor location and subtype. Lesions in critical areas (e.g., eyelids) or verrucous subtypes respond poorly, often requiring adjunct therapies (e.g., cryotherapy), which increase costs. Adverse reactions (edema, abscesses) occur in 30% of cases, necessitating additional veterinary care. Despite these challenges, large-scale cost–benefit analyses confirm BCG’s economic advantage over cisplatin, especially in herds with multiple affected animals, where reduced cumulative recurrence offsets side-effect management costs. Cisplatin requires specialized training for safe handling, while BCG can be administered by veterinarians with basic training using simplified protocols (e.g., 0.1–0.5 mg per lesion). This accessibility aligns BCG with One Health principles, promoting equitable solutions in low-resource settings [47,48,49,50,51].

## 9. Future Directions, Gaps, and Novel Insights

Despite decades of clinical use, Bacillus Calmette–Guérin (BCG) immunotherapy for equine sarcoids continues to be limited by protocol heterogeneity, lack of randomized controlled trials (RCTs), absence of predictive biomarkers, and insufficient long-term follow-up data [11,18,52]. These gaps restrict the ability to standardize treatment protocols and to identify which horses or lesion types are most likely to respond favorably. Furthermore, the mechanistic understanding of BCG-induced immune modulation in sarcoids remains incomplete, particularly regarding the dynamics of local immune cell infiltration, cytokine release patterns, and tumor microenvironment remodeling.

To enhance clinical comparability, consensus guidelines should be developed, including recommendations for standardized BCG dosing (0.1–0.5 mg per lesion), injection intervals (2–3 weeks), and careful selection of viable strains, ideally supported by multicentric randomized controlled trials. Such harmonization would improve reproducibility, facilitate meta-analyses, and provide clinicians with evidence-based therapeutic roadmaps [8,9]. Another critical research priority is the identification of predictive biomarkers—such as cytokine expression profiles, tumor-infiltrating lymphocyte density, or BPV oncogene transcript levels—that could help stratify patients and forecast responsiveness to BCG. Integration of omics-based platforms (genomics, proteomics, and immunomics) will be essential to refine personalized immunotherapy in equine oncology [22,23].

Future research should prioritize the design of prospective, controlled clinical trials with standardized protocols, systematic monitoring of immune responses, and long-term outcome assessment. Such studies are critical to establishing robust evidence for BCG efficacy and safety in equine oncology. In parallel, molecular and omics-based approaches (genomics, transcriptomics, proteomics, and immunomics) hold promise for identifying predictive biomarkers that can guide personalized immunotherapy in horses [53].

Beyond BCG, innovative immunotherapeutic strategies may redefine the landscape of sarcoid management. Checkpoint inhibition (e.g., PD-1/PD-L1 and CTLA-4 blockade) has demonstrated efficacy in human papillomavirus (HPV)-associated cancers and may be adapted for veterinary applications. CRISPR-Cas9-mediated editing of viral oncogenes (e.g., BPV E5) offers a potential curative approach, disrupting viral oncogenesis at its source. Nanoparticle-based delivery systems could enhance the precision, safety, and efficacy of immunomodulators or gene-editing tools in equine oncology [26,27,54].

**Figure 5:**
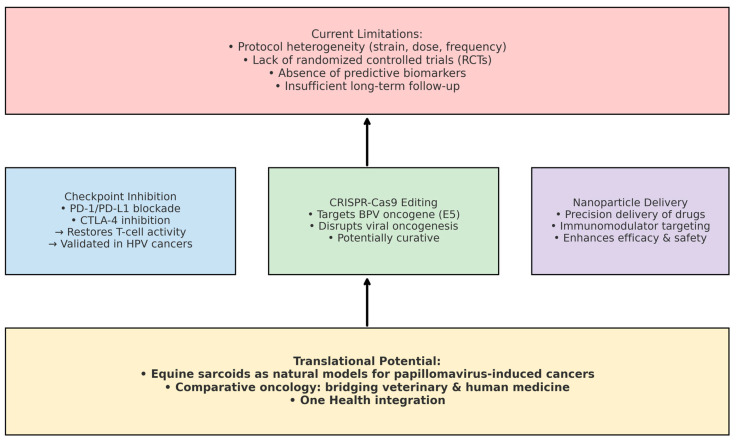
Future directions in equine sarcoid therapy. Current limitations include protocol heterogeneity, lack of randomized controlled trials, absence of predictive biomarkers, and insufficient long-term follow-up. Innovative strategies encompass checkpoint inhibition (PD-1/PD-L1, CTLA-4 blockade), CRISPR-Cas9-mediated disruption of BPV oncogenes, and nanoparticle-based delivery systems. These approaches highlight the translational potential of equine sarcoids as natural models for papillomavirus-induced cancers, bridging veterinary oncology with comparative oncology and One Health perspectives.

These innovations, combined with comparative oncology perspectives, position equine sarcoids as a natural model for papillomavirus-induced cancers, bridging veterinary and human medicine under a One Health paradigm [55,56]. By addressing current gaps and leveraging novel technologies, equine sarcoid therapy may advance from empirical immunotherapy to a data-driven, mechanistically informed field of translational oncology. A conceptual overview of these future directions is presented in Figure 5.

## 10. Conclusions

BCG immunotherapy remains a historically relevant yet clinically limited strategy for equine sarcoids. Despite decades of application, its inconsistent efficacy, protocol heterogeneity, and adverse inflammatory reactions have led to its decline compared with modern alternatives. Nevertheless, equine sarcoids provide a unique natural model of papillomavirus-induced oncogenesis, bridging veterinary and human oncology. Future progress requires rigorously designed randomized trials, standardized treatment protocols, and integration of novel strategies such as immune checkpoint inhibitors, CRISPR-Cas9 gene editing, and nanoparticle-based delivery systems. Critically, reframing equine sarcoid research within a comparative oncology and One Health context may yield translational benefits that extend beyond veterinary medicine.

## Figures and Tables

**Table 1:** Comparative analysis of efficacy, cost–benefit, and practical considerations in equine sarcoid therapies.

Treatment	Efficacy	Recurrence	Sessions	Cost (Relative)	Accessibility	Key Limitations
BCG Immunotherapy	60–70%	15–30%	1–3	Low (10× cheaper than cisplatin)	High (basic training needed)	Adverse reactions (30%)
Topical Cisplatin	70–85%	20–40%	4–6	High	Low (requires biosafety protocols)	Toxicity, logistical complexity
Surgical Excision	50–80%	20–40%	1	Moderate	Moderate (needs infrastructure)	High recurrence, postoperative care
Cryotherapy	60–75%	15–35%	2–4	Moderate	Low (requires specialized equipment)	Limited to accessible lesions

## Data Availability

The original contributions presented in the study are included in the article; further inquiries can be directed to the corresponding author.

## Supplementary Materials

The following supporting information can be downloaded at: https://www.mdpi.com/article/10.3390/v17101322/s1, Figure S1: Mixed verrucous and fibroblastic sarcoid affecting the upper eyelid of a 10-year-old Lusitano horse prior to intralesional BCG immunotherapy. The nodular lesion measured 9 cm (length) × 5 cm (width) × 3 cm (depth), with marked proliferative and ulcerated characteristics.; Figure S2: A and B: Upper eyelid of the same horse showing partial regression of the sarcoid lesion 21 days after the first intralesional BCG injection. C: Further reduction in lesion size observed 63 days after treatment initiation (third BCG application). D: Complete remission of the lesion at the conclusion of the therapeutic protocol.
