# Supplementary material for: BCG Immunotherapy in Equine Sarcoid Treatment: Mechanisms, Clinical Efficacy, and Challenges in Veterinary Oncology
Source: Viruses. 2025 Sep 29;17(10):1322. doi: 10.3390/v17101322 (PMC12567874; doi:10.3390/v17101322)
Supplement: Supplementary file 1 [file viruses-17-01322-s001.zip › viruses-3893694-supplementary/Supplementary Materials (Manuscritp ID viruses -3893694).pdf]

CERTIFICADO

Certificamos que a proposta intitulada "Estudo das doenças infecciosas, carenciais, metabólicas, genéticas e imunológicas em animais no Bioma Amazônico", protocolada sob o CEUA nº 6261300323 (ID 002208), sob a responsabilidade de **Felipe Masiero Salvarani** *e equipe; José Diomedes Barbosa Neto; Carlos Magno Chaves Oliveira ; Pedro Paulo Maia Teixeira; Natalia da Silva e Silva Silveira* - que envolve a produção, manutenção e/ou utilização de animais pertencentes ao filo Chordata, subfilo Vertebrata (exceto o homem), para fins de pesquisa científica ou ensino - está de acordo com os preceitos da Lei 11.794 de 8 de outubro de 2008, com o Decreto 6.899 de 15 de julho de 2009, bem como com as normas editadas pelo Conselho Nacional de Controle da Experimentação Animal (CONCEA), e foi **aprovada** pela Comissão de Ética no Uso de Animais da Universidade Federal do Pará (CEUA/UFPA) na reunião de 27/04/2023.

We certify that the proposal "Study of infectious, deficiency, metabolic, genetic and immunological diseases in animals in the Amazon Biome", utilizing 20 Bovines (males and females), 20 Buffalos (males and females), 20 Caprines (males and females), 20 Equines (males or females), 20 Ovines (males and females), protocol number CEUA 6261300323 (ID 002208), under the responsibility of **Felipe Masiero Salvarani** *and team; José Diomedes Barbosa Neto; Carlos Magno Chaves Oliveira ; Pedro Paulo Maia Teixeira; Natalia da Silva e Silva Silveira* - which involves the production, maintenance and/or use of animals belonging to the phylum Chordata, subphylum Vertebrata (except human beings), for scientific research purposes or teaching - is in accordance with Law 11.794 of October 8, 2008, Decree 6899 of July 15, 2009, as well as with the rules issued by the National Council for Control of Animal Experimentation (CONCEA), and was **approved** by the Ethic Committee on Animal Use of the Federal University of Para (CEUA/UFPA) in the meeting of 04/27/2023.

Finalidade da Proposta: Pesquisa

Vigência da Proposta: de 05/2023 a 04/2025 Área: Medicina Veterinária

Origem:	Não se aplica						
Espécie:	Bovinos	sexo:	Machos e Fêmeas	idade:	01 a 96 meses	N:	20
Linhagem:	Não se aplica			Peso:	15 a 700 kg		
Origem:	Não se aplica						
Espécie:	Bubalinos	sexo:	Machos e Fêmeas	idade:	01 a 96 meses	N:	20
Linhagem:	Não se aplica			Peso:	15 a 700 kg		
Origem:	Não se aplica						
Espécie:	Caprinos	sexo:	Machos e Fêmeas	idade:	01 a 72 meses	N:	20
Linhagem:	Não se aplica			Peso:	5 a 50 kg		
Origem:	Não se aplica						
Espécie:	Equídeos	sexo:	Machos ou Fêmeas	idade:	01 a 96 meses	N:	20
Linhagem:	Não se aplica			Peso:	15 a 700 kg		
Origem:	Não se aplica						
Espécie:	Ovinos	sexo:	Machos e Fêmeas	idade:	01 a 72 meses	N:	20
Linhagem:	Não se aplica			Peso:	5 a 50 kg		

Local do experimento: Os animais a serem utilizados no experimento serão oriundos de diferentes propriedades e produtores no Estado do Pará e todos as propriedades serão cadastradas por meio de fichas clínicas do Hospital Veterinário do Instituto de Medicina Veterinária da UFPA/Castanhal e todos os proprietários terão que preencher o termo TERMO DE CONSENTIMENTO LIVRE E ESCLARECIMENTO PARAE PERMISSÃO PARA COLETAS E UTILIZAÇÃO DE DADOS

Belém, 26 de julho de 2023

Profa. Dra. Barbarella de Matos Macchi
Coordenadora da Comissão de Ética no Uso de Animais
Universidade Federal do Pará

Prof. Dr. James Tony Lee
Vice-Coordenador da Comissão de Ética no Uso de Animais
Universidade Federal do Pará

PRISMA 2020 Checklist

Section and Topic	Item #	Checklist item	Location where item is reported
**TITLE**			
Title	1	Identify the report as a systematic review.	Yes
**ABSTRACT**			
Abstract	2	See the PRISMA 2020 for Abstracts checklist.	Yes
**INTRODUCTION**			
Rationale	3	Describe the rationale for the review in the context of existing knowledge.	Yes
Objectives	4	Provide an explicit statement of the objective(s) or question(s) the review addresses.	Yes
**METHODS**			
Eligibility criteria	5	Specify the inclusion and exclusion criteria for the review and how studies were grouped for the syntheses.	Yes
Information sources	6	Specify all databases, registers, websites, organisations, reference lists and other sources searched or consulted to identify studies. Specify the date when each source was last searched or consulted.	Yes
Search strategy	7	Present the full search strategies for all databases, registers and websites, including any filters and limits used.	Yes
Selection process	8	Specify the methods used to decide whether a study met the inclusion criteria of the review, including how many reviewers screened each record and each report retrieved, whether they worked independently, and if applicable, details of automation tools used in the process.	Yes
Data collection process	9	Specify the methods used to collect data from reports, including how many reviewers collected data from each report, whether they worked independently, any processes for obtaining or confirming data from study investigators, and if applicable, details of automation tools used in the process.	Yes
Data items	10a	List and define all outcomes for which data were sought. Specify whether all results that were compatible with each outcome domain in each study were sought (e.g. for all measures, time points, analyses), and if not, the methods used to decide which results to collect.	Yes
	10b	List and define all other variables for which data were sought (e.g. participant and intervention characteristics, funding sources). Describe any assumptions made about any missing or unclear information.	Yes
Study risk of bias assessment	11	Specify the methods used to assess risk of bias in the included studies, including details of the tool(s) used, how many reviewers assessed each study and whether they worked independently, and if applicable, details of automation tools used in the process.	Yes
Effect measures	12	Specify for each outcome the effect measure(s) (e.g. risk ratio, mean difference) used in the synthesis or presentation of results.	Yes
Synthesis methods	13a	Describe the processes used to decide which studies were eligible for each synthesis (e.g. tabulating the study intervention characteristics and comparing against the planned groups for each synthesis (item #5)).	Yes
	13b	Describe any methods required to prepare the data for presentation or synthesis, such as handling of missing summary statistics, or data conversions.	Yes
	13c	Describe any methods used to tabulate or visually display results of individual studies and syntheses.	Yes
	13d	Describe any methods used to synthesize results and provide a rationale for the choice(s). If meta-analysis was performed, describe the model(s), method(s) to identify the presence and extent of statistical heterogeneity, and software package(s) used.	Yes
	13e	Describe any methods used to explore possible causes of heterogeneity among study results (e.g. subgroup analysis, meta-regression).	Yes
	13f	Describe any sensitivity analyses conducted to assess robustness of the synthesized results.	Yes
Reporting bias assessment	14	Describe any methods used to assess risk of bias due to missing results in a synthesis (arising from reporting biases).	Yes
Certainty assessment	15	Describe any methods used to assess certainty (or confidence) in the body of evidence for an outcome.	Yes

PRISMA

PRISMA 2020 Checklist

Section and Topic	Item #	Checklist item	Location where item is reported
**RESULTS**			
Study selection	16a	Describe the results of the search and selection process, from the number of records identified in the search to the number of studies included in the review, ideally using a flow diagram.	Yes
	16b	Cite studies that might appear to meet the inclusion criteria, but which were excluded, and explain why they were excluded.	Yes
Study characteristics	17	Cite each included study and present its characteristics.	Yes
Risk of bias in studies	18	Present assessments of risk of bias for each included study.	Yes
Results of individual studies	19	For all outcomes, present, for each study: (a) summary statistics for each group (where appropriate) and (b) an effect estimate and its precision (e.g. confidence/credible interval), ideally using structured tables or plots.	Yes
Results of syntheses	20a	For each synthesis, briefly summarise the characteristics and risk of bias among contributing studies.	Yes
	20b	Present results of all statistical syntheses conducted. If meta-analysis was done, present for each the summary estimate and its precision (e.g. confidence/credible interval) and measures of statistical heterogeneity. If comparing groups, describe the direction of the effect.	Yes
	20c	Present results of all investigations of possible causes of heterogeneity among study results.	Yes
	20d	Present results of all sensitivity analyses conducted to assess the robustness of the synthesized results.	Yes
Reporting biases	21	Present assessments of risk of bias due to missing results (arising from reporting biases) for each synthesis assessed.	Yes
Certainty of evidence	22	Present assessments of certainty (or confidence) in the body of evidence for each outcome assessed.	Yes
**DISCUSSION**			
Discussion	23a	Provide a general interpretation of the results in the context of other evidence.	Yes
	23b	Discuss any limitations of the evidence included in the review.	Yes
	23c	Discuss any limitations of the review processes used.	Yes
	23d	Discuss implications of the results for practice, policy, and future research.	Yes
**OTHER INFORMATION**			
Registration and protocol	24a	Provide registration information for the review, including register name and registration number, or state that the review was not registered.	Yes
	24b	Indicate where the review protocol can be accessed, or state that a protocol was not prepared.	Yes
	24c	Describe and explain any amendments to information provided at registration or in the protocol.	Yes
Support	25	Describe sources of financial or non-financial support for the review, and the role of the funders or sponsors in the review.	Yes
Competing interests	26	Declare any competing interests of review authors.	Yes
Availability of data, code and other materials	27	Report which of the following are publicly available and where they can be found: template data collection forms; data extracted from included studies; data used for all analyses; analytic code; any other materials used in the review.	Yes

From: Page MJ, McKenzie JE, Bossuyt PM, Boutron I, Hoffmann TC, Mulrow CD, et al. The PRISMA 2020 statement: an updated guideline for reporting systematic reviews. BMJ 2021;372:n71. doi: 10.1136/bmj.n71.
This work is licensed under CC BY 4.0. To view a copy of this license, visit https://creativecommons.org/licenses/by/4.0/

Supplementary Materials

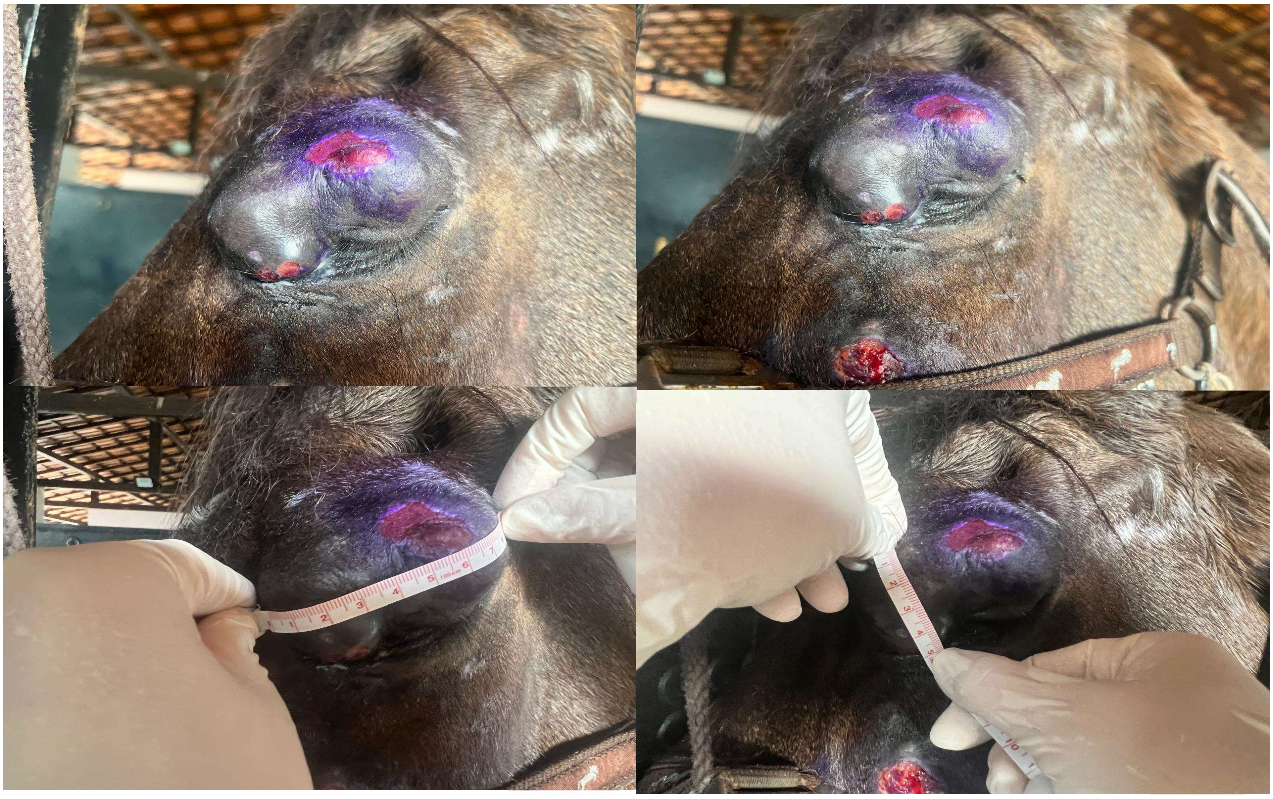

Figure S1: Mixed verrucous and fibroblastic sarcoid affecting the upper eyelid of a 10-year-old Lusitano horse prior to intralesional BCG immunotherapy. The nodular lesion measured 9 cm (length) × 5 cm (width) × 3 cm (depth), with marked proliferative and ulcerated characteristics.

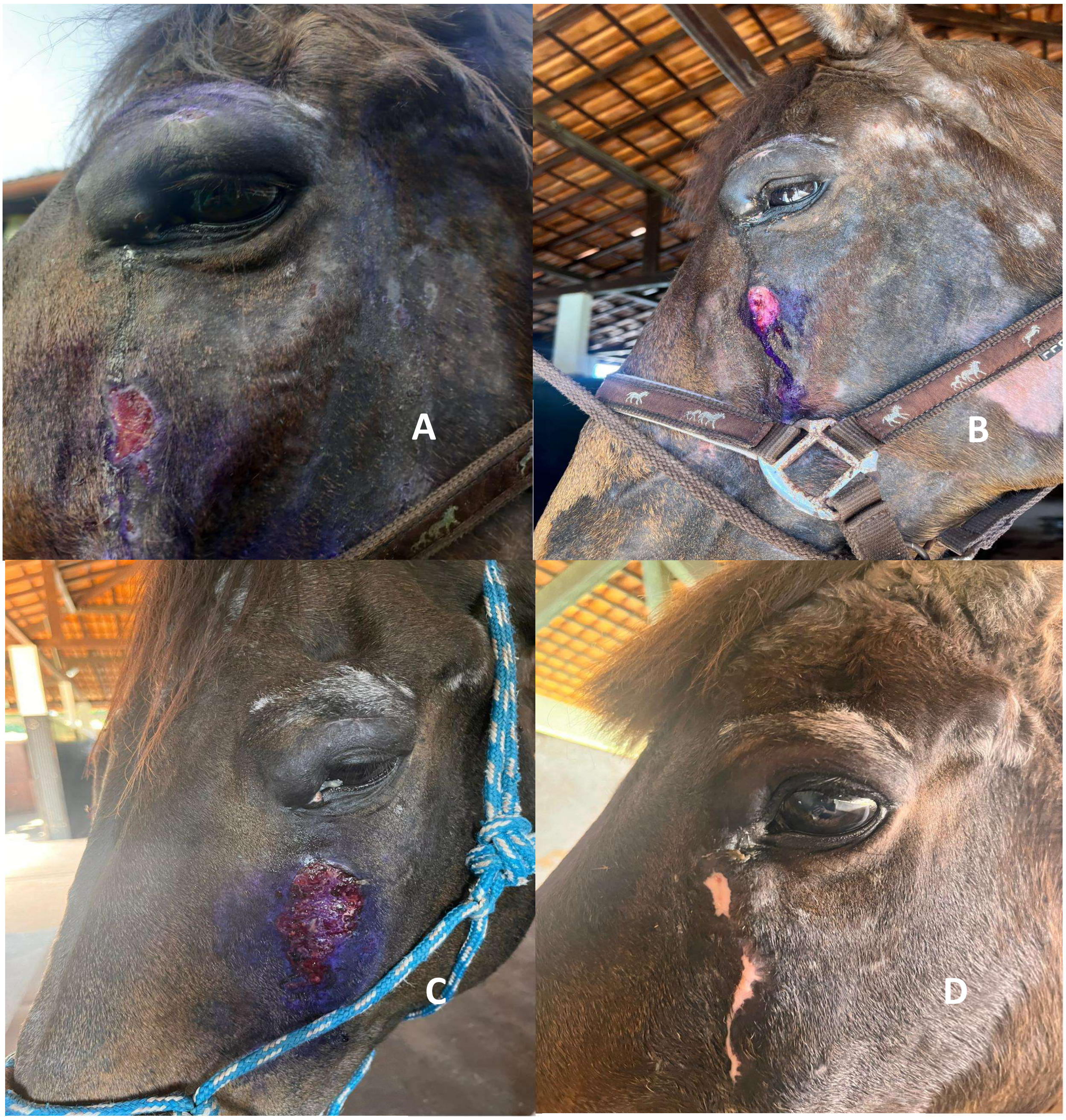

Figure S2: A and B: Upper eyelid of the same horse showing partial regression of the sarcoid lesion 21 days after the first intralesional BCG injection. C: Further reduction in lesion size observed 63 days after treatment initiation (third BCG application). D: Complete remission of the lesion at the conclusion of the therapeutic protocol.
