# Supplementary figures and images for: BCG Immunotherapy in Equine Sarcoid Treatment: Mechanisms, Clinical Efficacy, and Challenges in Veterinary Oncology
Source: Viruses. 2025 Sep 29;17(10):1322. doi: 10.3390/v17101322 (PMC12567874; doi:10.3390/v17101322)

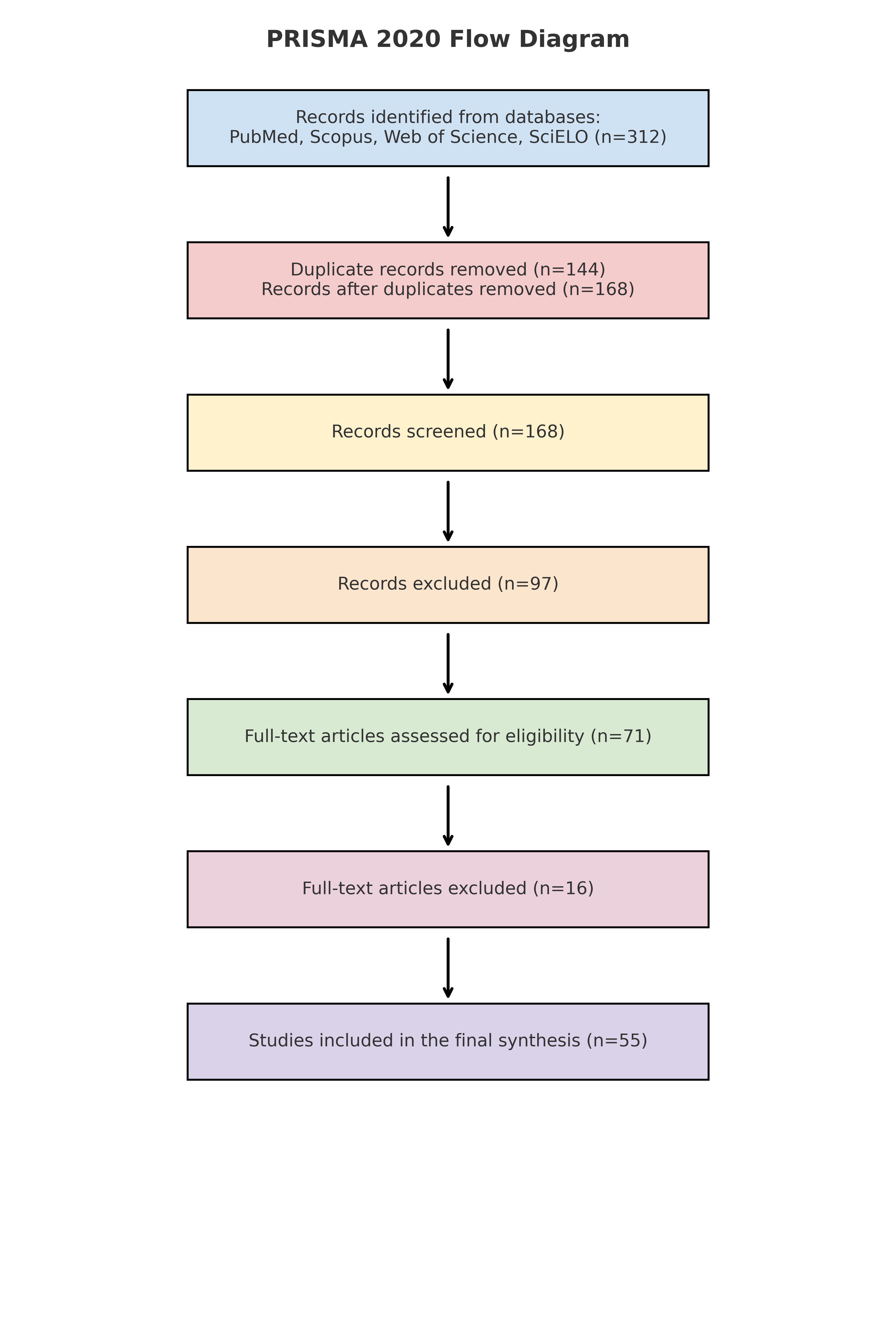

Supplement: Supplementary file 1 [file viruses-17-01322-s001.zip › viruses-3893694-supplementary/PRISMA_Flow_Diagram_Equine_Sarcoids .tiff]
